# Novel Safranin-Tinted *Candida rugosa* Lipase Nanoconjugates Reagent for Visualizing Latent Fingerprints on Stainless Steel Knives Immersed in a Natural Outdoor Pond

**DOI:** 10.3390/ijms19061576

**Published:** 2018-05-25

**Authors:** Aida Rasyidah Azman, Naji Arafat Mahat, Roswanira Abdul Wahab, Fazira Ilyana Abdul Razak, Hafezul Helmi Hamzah

**Affiliations:** 1 Department of Chemistry, Faculty of Science, Universiti Teknologi Malaysia, Skudai 81310, Malaysia; aidarasyidah@rocketmail.com (A.R.A.); fazirailyana@utm.my (F.I.A.R.); 2 Criminal Investigation Department, Criminal Intelligence (D4), Royal Malaysia Police, Kuala Lumpur 50560, Malaysia; fezulelmihamzah@gmail.com

**Keywords:** forensic science, latent fingerprints, non-porous objects, immersion, natural outdoor pond, *Candida rugosa* lipase nanoconjugates, nanobio-based method

## Abstract

Waterways are popular locations for the disposition of criminal evidence because the recovery of latent fingerprints from such evidence is difficult. Currently, small particle reagent is a method often used to visualize latent fingerprints containing carcinogenic and hazardous compounds. This study proposes an eco-friendly, safranin-tinted *Candida rugosa* lipase (triacylglycerol ester hydrolysis EC 3.1.1.3) with functionalized carbon nanotubes (CRL-MWCNTS/GA/SAF) as an alternative reagent to the small particle reagent. The CRL-MWCNTS/GA/SAF reagent was compared with the small particle reagent to visualize groomed, full fingerprints deposited on stainless steel knives which were immersed in a natural outdoor pond for 30 days. The quality of visualized fingerprints using the new reagent was similar (modified-Centre for Applied Science and Technology grade: 4; *p* > 0.05) to small particle reagent, even after 15 days of immersion. Despite the slight decrease in quality of visualized fingerprints using the CRL-MWCNTS/GA/SAF on the last three immersion periods, the fingerprints remained forensically identifiable (modified-Centre for Applied Science and Technology grade: 3). The possible chemical interactions that enabled successful visualization is also discussed. Thus, this novel reagent may provide a relatively greener alternative for the visualization of latent fingerprints on immersed non-porous objects.

## 1. Introduction

The uniqueness of fingerprints is attributed to their individual characteristics, persistency, and systematic classifications of general ridge patterns [1]. Accordingly, the use of fingerprints is important as forensic evidence for human identification, especially during crime investigations [2]. Although visible fingerprints are those prints that are left on transferable, colored media (e.g., ink, blood, and grease), plastic fingerprints refer to the impressions left on soft objects (e.g., putty, wax, and soap) [1]. Latent fingerprints are also prints that are hidden to the naked eye and, accordingly, require the use of visualization methods [1]. Fingerprints are composed of a mixture of natural secretions from eccrine and sebaceous glands as well as extrinsic constituents, e.g., bacteria spores, dust, and cosmetics [3]. Eccrine glands secrete water (comprising more than 90% of gland secretions) as well as organic (e.g., amino acids) and inorganic (e.g., sodium and potassium) compounds [3]. Conversely, sebaceous glands primarily secrete lipids such as fatty acids and squalene [3,4,5]. 

The recovery of potential forensic evidence such as fingerprints during underwater investigations is often a challenging task for forensic investigators [6] as the water-soluble constituents of latent fingerprints are easily washed off by water. Numerous methods have been proposed to visualize latent fingerprints on wet objects. Among many others, Beresford and Hillman [7] introduced the spatially selective deposition of electrochromic polymer (polyaniline) for visualizing the inter-ridge of a fingerprint. This technique generates a negative impression of the visualized fingerprints on stainless-steel plates. Similarly, electrochromic copolymer films (pyrrole and 3,4-ethylenedioxythiophene) were reportedly employed to generate negative impression of the visualized fingerprints on stainless steel plates [8]. Small particle reagent (SPR) is a popular method which has been widely used for visualizing latent fingerprints on wet non-porous objects [1,9], presumably capitalizing on any non-water-soluble fingerprint constituents [9]. Notably, SPR utilizes a suspension of fine particles of either titanium dioxide [10] or molybdenum disulphide [11] in a surfactant. However, such contrasting agents can be hazardous not only to humans but also to the environment, especially over prolonged exposure. Although titanium dioxide is both potentially carcinogenic [10,12], attributable to the excessive production of intracellular reactive oxygen species [13], and environmentally toxic [14], prolonged exposure to molybdenum has been associated with chronic respiratory effects as well as irritation of the eyes, nose, and skin [15]. Such possible health risks can be amplified, especially when a SPR solution is prepared by lab technicians. In addition, the combination of molybdenum and other environmental stressors have been found to cause substantial declines in aquatic fauna populations, such as the juvenile kokanee salmon [16]. It has been reported that the concentrations of elements ‘in effluents are often elevated above background seawater’ [17]. Additionally, the use of SPR requires washing, which may lead to the continuous buildup of toxic metals. Therefore, its potential threat towards human as well as the marine environment cannot be neglected. In view of this, the development of suitable greener alternatives, such as the use of enzymes, may prove necessary.

Lipases (triacylglycerol ester hydrolysis EC 3.1.1.3) are important biomolecules for various chemical reactions, attributable to their high activity and broad specificity in reaction mediums [18]. Such advantages have resulted in widespread popularity of lipases at the industrial level [19]. As one of the most versatile biocatalysts, the use of *Candida rugosa* lipase (CRL) for catalyzing reactions, e.g., esterification and transesterification, has been extensively reported [20,21]. In this context, the use of multi-walled carbon nanotubes (MWCNTs) as nanosupports has also been suggested for enhancing activity and stability as well as for extending the reaction life of CRL [21]. Interestingly, although the application of lipases has been commonplace in various bioindustries [22], its utilization for visualizing latent fingerprints on immersed objects for forensic identification remains unreported thus far.

For the first time, this paper explores the feasibility of using safranin-tinted CRL nanoconjugates reagent (CRL immobilized onto MWCNTs, glutaraldehyde (GA) and safranin T (SAF)) (CRL-MWCNTs/GA/SAF) for visualizing latent fingerprints on stainless steel knives. The knives were immersed for 30 consecutive days in a natural outdoor pond and then visualized using the CRL-MWCNTs/GA/SAF reagent. The study also used computational chemistry to evaluate the possible chemical interactions that occurred between the lipid constituents of the fingerprints and that of CRL-MWCNTs/GA/SAF reagent, enabling the successful visualization of the latent fingerprints. A comparison of the quality of visualized fingerprints using this newly developed safranin-tinted CRL nanoconjugates reagent with that of SPR was made following the modified grading scheme of Centre for Applied Science and Technology (m-CAST), suggested by Bandey and Gibson [23].

## 2. Results and Discussion

### 2.1. Proposed Chemical Interactions

To improve the bioactivity of MWCNTs, purification using HCl was carried out to eliminate non-polarizable compounds (e.g., impurities and unreacted reagents) [24], amorphous carbon, and residual metal [25] on the surface and sidewalls of raw tubular MWCNTs. Notably, purification was performed to prime the tubular sidewalls with numerous reaction sites, thus increasing sidewall functionalization of the MWCNTs [25]. Upon functionalization of P-MWCNTs with a mixture of HNO_3_ and H_2_SO_4_ (1:3 *v*/*v*), polar carboxylate groups (COO–) were introduced to the surface and tubular sidewalls of MWCNTs. The carboxylate groups were intended to function as anchors (via hydrogen bond) to other polar groups (i.e., NH_2_ and OH) on the surface of the CRL [21]. 

The first step of the study comprised a visualization of latent fingerprints, which involved rinsing the then-immersed stainless steel knives with ultra-pure water. This step was required to wash off any dirt or mud that could impede the subsequent fingerprint visualization process. Additionally, the prerinsing step aided the attachment of CRL-MWCNTs onto the surface of the lipid. Current literature has shown that the activity/affinity of CRL-MWCNTs towards the lipid constituents of fingerprints is amplified at the lipid–water interface by a process called interfacial activation [26,27]. 

**Scheme 1:**
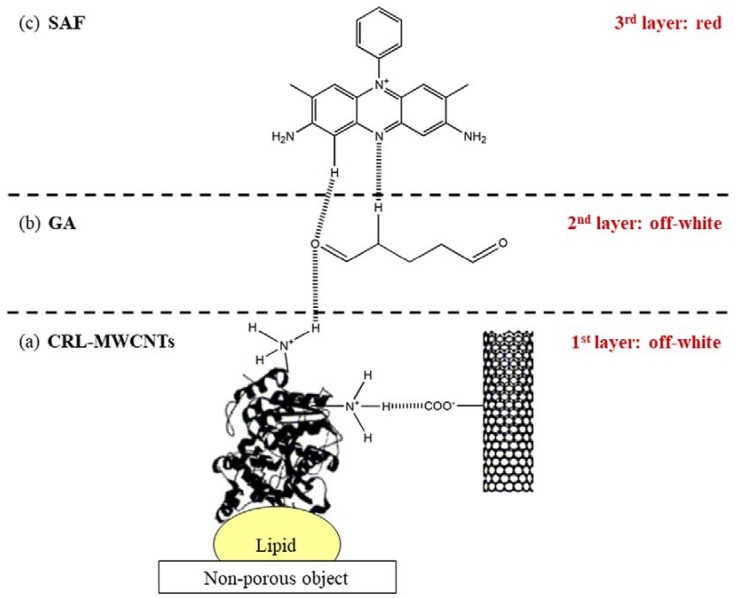
Schematic illustration of the visualization of latent fingerprint using the safranin-tinted *Candida rugosa* lipase-multi-walled carbon nanotubes/glutaraldehyde/safranin reagent (**a**–**c**).

The hypothesized layering of the safranin-tinted CRL nanoconjugates reagent onto the lipids of fingerprints is illustrated in Scheme 1a–c. The possible attachment of CRL-MWCNTs onto the lipids of fingerprints resulted in the appearance of an initial off-white layer on the fingerprint ridges (Scheme 1a), attributable to the high selectivity of CRL-MWCNTs. Results from computational chemistry analysis supported the formation of hydrogen bonds between the GA and -NH_2_ moieties (from lysine, arginine, histidine) on the surface of the CRL (Scheme 1b), following the spraying of the GA onto the lipid-CRL-MWCNTs complex. This may have subsequently primed the surface of the treated fingerprints with suitable moieties (i.e., C=O groups) for attachment with the positively charged SAF molecules, as suggested by computational results. The additional layering with GA created an additional off-white layer on the fingerprint ridges, which was undetectable by the naked eye. 

To facilitate visualization and provide contrast for the fingerprint, SAF red dye was used to tint the lipid-CRL-MWCNTs/GA ridges. The study proposed that the adherence of SAF to CRL-MWCNTs/GA was via an interaction of the negatively charged C=O of GA with that of the positively charged SAF (Scheme 1c). Notably, the discussion on the proposed chemical interactions was made by only considering the results obtained during field emission scanning electron microscopy analysis and a computational chemistry approach. Therefore, further investigations using a molecular dynamics approach and transmission electron microscopic may prove useful for elucidating the real chemical interactions among the different constituents of the reagent with that of fingerprints.

### 2.2. Quality of the Visualized Fingerprints Using the Safranin-Tinted Candida rugosa Lipase Nanoconjugates Reagent

**Figure 1:**
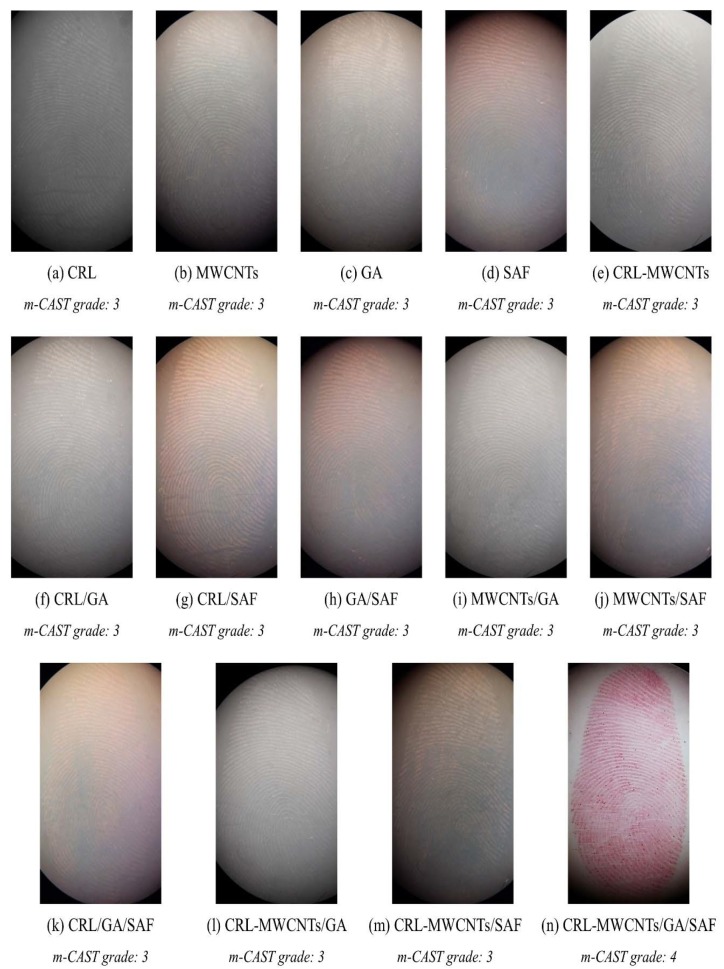
Visualization of latent fingerprints using various combinations of CRL, MWCNTs, GA and SAF observed at 7.5× magnification (**a**–**n**).

**Figure 2:**
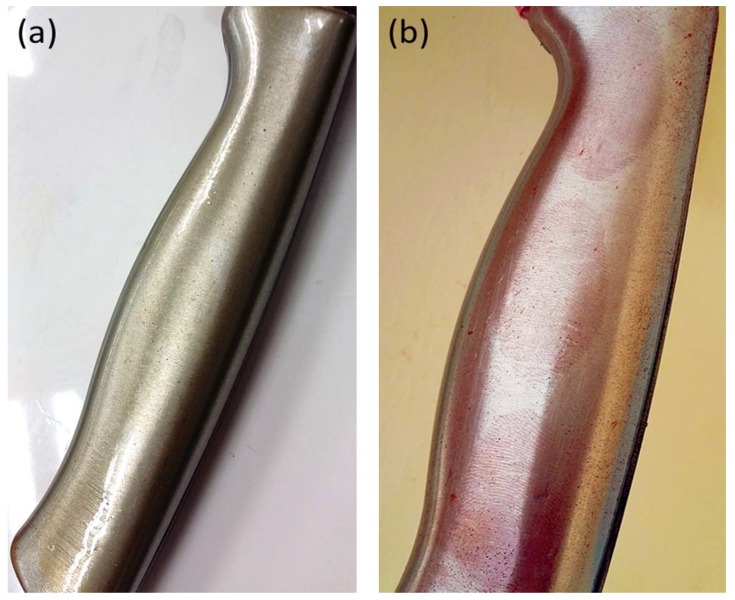
Representative photographs of fingerprints on a knife immersed in a natural outdoor pond (for five days) before (**a**) and after (**b**) the treatment with the safranin-tinted CRL nanoconjugates reagent.

It was observed that the quality and contrast of the fingerprint ridges were improved following the use of CRL-MWCNTs solution when compared with that of CRL and MWCNTs solutions separately in Figure 1a–n. Such an improvement may be attributable to the enhanced structural stability, activity, and selectivity [28] as well as excellent binding capacity and biological compatibility [29] of the immobilized enzymes, i.e., CRL-MWCNTs. The study noted that further improvements in the quality and contrast of the visualized fingerprints were observed upon treating the fingerprint-CRL-MWCNTs complexes with the GA and SAF solutions. Observations revealed that fingerprints visualized using the safranin-tinted CRL nanoconjugates reagent demonstrated the best quality and contrast when compared with other trials, as seen in Figure 1a–n. Accordingly, the use of GA as a cross-link between CRL-MWCNTs, SAF, and CRL-MWCNTs (as the biocatalyst) via counteraction with the lipid components of fingerprints provided successful visualization, rendering satisfactory forensic identification. Such a finding can be attributable to the fact that the short-chain amino acid constituents of fingerprints might have been washed off by water, leaving only the lipid constituents on the immersed objects [30]. In this study, the role of CRL, as the specific enzyme that interacted with the lipids [26], was integral in precisely tracing the lipid components and enabling visualization of the fingerprints. The activity/affinity of CRL-MWCNTs towards lipid constituents of fingerprints typically became amplified at the lipid-water interface by a peculiar but well-reported phenomenon for lipases called “interfacial activation” [26,27]. Under such conditions, the CRL becomes activated for interactions which are specific to lipids only; these activations follow the hydrophobicity of the lipid which induces the ‘lid opening’ phenomenon [31,32]. This process displaces a hydrophobic polypeptide loop over the catalytic tunnel and permits access of a substrate (attachment of lipid) into the catalytic site of the lipase [33]. Clearly, the CRL used in this research acts as a greener, lipid-specific natural surfactant to replace the chemical-based surfactant used in SPR formulation. Moreover, literature has shown that the utilization of the prevailing SPR can be associated with numerous human [10,12,13,15,34] and ecological toxicities [14,16,17,35]. Hence, this safranin-tinted CRL nanoconjugates reagent is a promising greener alternative to visualize latent fingerprints on non-porous objects that have been accidentally or deliberately wetted. Representative photographs of fingerprints on a knife immersed in a natural outdoor pond (for five days) before and after the treatment with the safranin-tinted CRL nanoconjugates reagent are presented in Figure 2a,b.

Notably, the SAF is a common dye used for food, textile [36], and biological applications [37], with high Lethal Dose-50 values before exerting toxicity to living organisms. It has been reported that the LD50 via inhalation in rabbits and rats is at 81,000 mg/m^3^/14 h and 64,000 ppm/4 h, respectively. The doses via oral ingestion for mouse, rabbits, and rats are 7300 mg/kg, 14,200 mg/kg and 5600 mg/kg, respectively. In addition, the LD50 via skin absorption for rabbits is 15,800 mg/kg. Following the Draize eye test for rabbits, moderate irritation to the eyes and skin were observed at 100 mg/24 h and 20 mg/24 h, respectively [38]. A review of current literature revealed no lethality in humans that can be exclusively attributed to SAF toxicity. Although this research used SAF as a dye for enhancing the contrast of the visualized latent fingerprints, the prepared concentration was very low, at only 1 g of SAF in 20 mL of ultra-pure water. The prepared concentration of SAF was sufficient for staining 40 fingerprints, bringing the amount of SAF required for each fingerprint to 25 mg. Because the study used a concentration of SAF that was considerably lower than the reported LD50s [38], it can be inferred that a prepared CRL-MWCNTs/GA/SAF formulation is less likely to incur adverse reactions in humans following exposure at that concentration.

### 2.3. Comparison on the Quality of Visualized Fingerprints Using the Safranin-Tinted CRL Nanoconjugates Reagent with That of Small Particle Reagent

**Figure 3:**
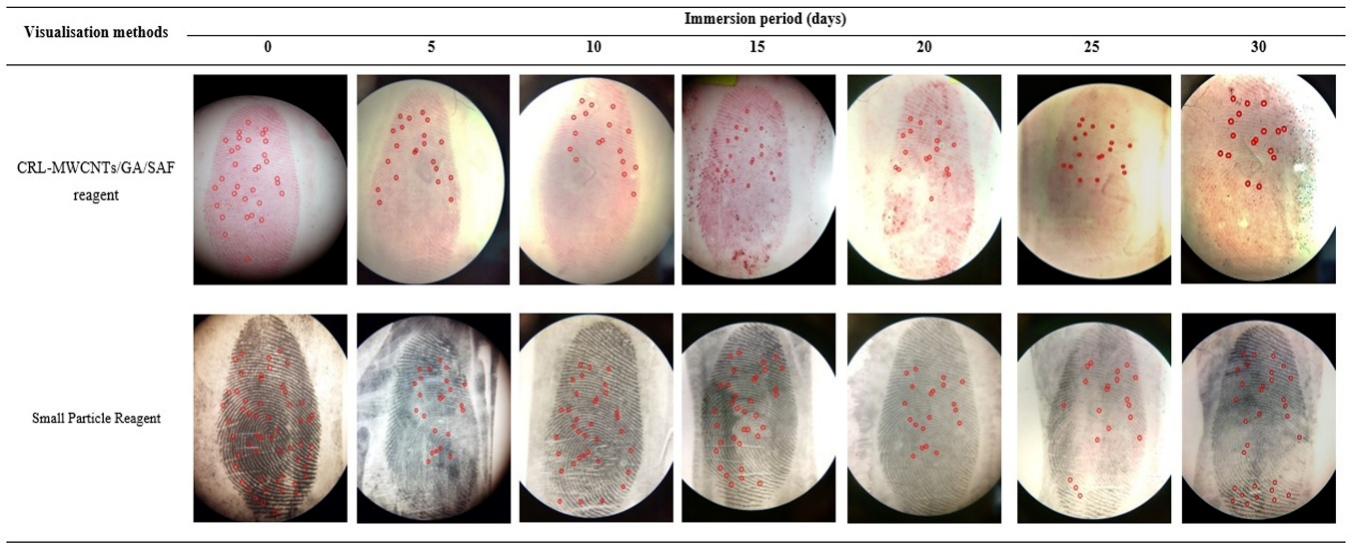
Visualized fingerprints on stainless steel knives immersed in a natural outdoor pond for specific immersion periods using the safranin-tinted CRL nanoconjugates reagent (**above**) and SPR (**below**) observed at 7.5× magnification. The red dots represent the minutiae count of fingerprints for forensic identification purposes.

The quality of visualized fingerprints on knives immersed in a natural outdoor pond for up to 30 days is presented in Table 1 and Figure 3. Interestingly, despite complete exposure to a multitude of uncontrollable outdoor environmental conditions of the natural outdoor pond water, the quality of fingerprints visualized using both reagents was remarkably good (m-CAST grade: 3–4), even at 30 days of immersion (Table 1, Figure 3). The m-CAST grade 4 signifies “full development—whole mark clear with continuous ridges”, while m-CAST grade 3 refers to “>2/3 of mark with continuous ridges, but not quite a perfect mark” [23]. Comparable to the quality observed for SPR, the visualized latent fingerprints using the safranin-tinted CRL nanoconjugates reagent were of m-CAST grade 4 for up to 15 days of immersion in the natural outdoor pond. Beyond this duration, a slight decrease in the quality (m-CAST grade 3) of visualized latent fingerprints was observed using the safranin-tinted CRL nanoconjugates reagent (Table 1, Figure 3).

To investigate the identifiability of the visualized fingerprints for forensic practical casework, the lifted fingerprints were submitted to the Central Criminal Registry for Malaysia and Singapore, located at the Royal Malaysia Police (RMP) Headquarters, Bukit Aman. A fingerprint expert examined the fingerprints visualized using both the safranin-tinted CRL nanoconjugates reagent and that of SPR using the 12-point matched minutiae standard used in Malaysia [39]. The results revealed that all the fingerprints visualized by both methods had sufficient ridge characteristics (minutiae) to enable forensic identification. 

Considerable degradation in the quality of visualized fingerprints on objects submerged in stagnant waters in laboratory has been previously reported in the literature [9,30]. Interestingly, although the contrast of the fingerprints visualized using both reagents was observably reduced over time (Figure 3), the quality reduction of the visualized fingerprints appeared trivial. Notably, the fingerprints remained identifiable even at 30 days of immersion in the natural outdoor pond water. Because the safranin-tinted CRL nanoconjugates reagent detects the presence of lipid constituents of fingerprints, the degradation in contrast and quality of the visualized fingerprints following a longer duration of exposure can be attributed to microbial activity that hydrolyzed the majority of the lipid component of the fingerprints [9]. Another contributing factor was the mechanical effect of pond water that further exacerbated the degradation process of the lipid constituents. As chemical optimization was not attempted at this stage, the use of Response Surface Methodology (RSM) may prove necessary for optimizing the reagent to improve its performance.

### 2.4. Characterization of Visualized Fingerprints Using the Safranin-Tinted CRL Nanoconjugates Reagent

#### 2.4.1. Characterizations of Visualized Fingerprints Using Field Emission Scanning Electron Microscope (FESEM)

**Figure 4:**
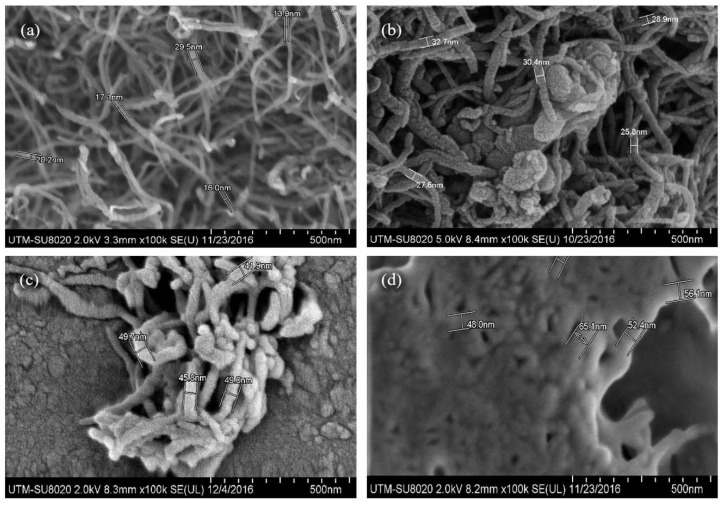
Field emission scanning electron microscopy photomicrographs of (**a**) F-MWCNTs as well as (**b**) CRL-MWCNTs, (**c**) CRL-MWCNTs/GA, and (**d**) CRL-MWCNTs/GA/SAF solutions on a fingerprint.

Although successful visualization of latent fingerprints using the safranin-tinted CRL nanoconjugates reagent was observed, the nanoscale morphological assessment using the Field Emission Scanning Electron Microscope (FESEM) offered better empirical evidence to elucidate the attachment of the nanobiocomposite on the latent fingerprints. The morphological features of the functionalized-MWCNTs (F-MWCNTs) as well as CRL-MWCNTs, CRL-MWCNTs/GA, and CRL-MWCNTs/GA/SAF solutions deposited on the lipid of the fingerprint are presented in Figure 4a–d. The micrograph showed that the diameters of F-MWCNTs increased, from a range of 13.9–29.5 nm (Figure 4a) to 25.8–32.7 nm upon immobilization of CRL onto F-MWCNTs (CRL-MWCNTs) (Figure 4b), which inferred the possible deposition of CRL onto the F-MWCNTs. This finding was also supported by previously reported studies which utilized F-MWCNTs as nanosupports for CRL [20,21]. Correspondingly, the thickening of the exterior of the CRL-MWCNTs corroborated the presence of CRL deposits on the surface of F-MWCNTs, likely attributable to the “evenness of the enzyme ‘coating’” over the nanotubes [40]. The morphological change was consistent with a previous report which argued that lipases tended to form “bimolecular aggregates” on functionalized surfaces [41]; this finding agreed with the increased diameter of the CRL-MWCNTs seen in this study. Moreover, attachment of the cross-linker GA (Figure 4c) with the polar amino groups on the exterior of the CRL-MWCNTs formed an additional layer on the lipid-CRL-MWCNTs. The presence of an additional layer of GA was supported by further diameter increase of the formed CRL-MWCNTs/GA (41.9–49.7 nm). This indicated successful attachment of the cross-linker onto the CRL-MWCNTs. Subsequently, the diameter of CRL-MWCNTs/GA was further increased from an average of 48.0 nm to 65.1 nm (Figure 4d) when the SAF solution was sprayed onto the lipid-CRL-MWCNTs/GA deposits. Thus, the final reddish-tinted fingerprint, now visible to the naked eye, was an indication of the attachment of the negatively charged C=O of GA onto the positively charged SAF molecules.

#### 2.4.2. Computational Chemistry

**Figure 5:**
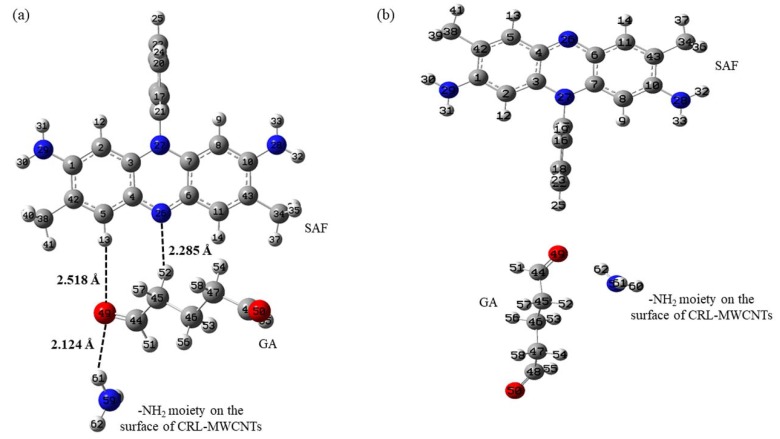
Conformations and atomic numbers for (**a**) structure A and the three observed hydrogen bonds as well as (**b**) structure B. Oxygen, hydrogen, nitrogen, and carbon atoms are marked in red, white, blue, and grey, respectively.

Two conformations of the complexes (labelled structures A and B) were considered in the present study, and its conformations as well as atomic numbers are shown in Figure 5a,b. The optimized bond lengths and angles were also calculated. For both conformations (Figure 5a,b), the C–C bond lengths were approximately 1.500 Å, close to the normal C–C single bond length reported in previous studies [42]. Moreover, the C48-O50 (1.237 Å) and C44-O49 (1.246 Å) bonds exhibited typical double bond characteristics. The bond lengths for C–C aromatic bonds were approximately 1.400 Å, which correlated well with previously reported values. As anticipated, the bond lengths for C6-N26 and C4-N26 were also about 1.376 Å, which fell well within the values for aromatic C–N [42]. The bond length for N–H was about 1.000 Å, and the bond angles were approximately 1200 for all aromatic rings. Therefore, it appears that the optimized results on bond lengths and angles corresponded to those reported by previous researchers [42], verifying the calculated results of the present study. The data indicated that structure A was the most stable for further in silico investigation to determine the hydrogen bonding interactions that formed the CRL-MWCNTs/GA/SAF complex.

The International Union of Pure and Applied Chemistry (IUPAC) defines a hydrogen bond as an attractive interaction between a hydrogen atom from a molecule or a molecular fragment X–H, with X being more electronegative than that of H, with evidence of bond formation [43]. According to this definition, the sum of the Van der Waals atomic radii of hydrogen and oxygen needs to be less than 2.500 Å to indicate the presence of an intramolecular hydrogen bond [44]. Although the bond values of <2.500 Å represent strong hydrogen bonds, values of >2.500 Å would indicate weak hydrogen bonds [45]. A computational analysis on structure A revealed the possible presence of a strong hydrogen bond (O49-H61, 2.124 Å) between –NH_2_ moiety, which was found on the surface of CRL-MWCNTs and GA molecules (Figure 5a). This newly formed hydrogen bond agreed with the observed increase in the diameter of CRL-MWCNTs in the FESEM micrographs, from 25.8–32.7 nm (Figure 4b) to 41.9–49.7 nm (Figure 4c) after treatment with GA. Furthermore, two possible hydrogen bonds (049-H13, 2.518 Å and N26-H52, 2.285 Å) were observed between GA and SAF molecules, with the N26-H52 being stronger than that of 049-H13 (Figure 5a). Accordingly, the data suggest that the SAF was present on the CRL-MWCNTs/GA. This was supported by the thickening effect of the CRL-MWCNTs/GA (~48.0 nm), which was observed in the FESEM micrograph after spraying with the SAF reagent, producing the reddish-tinted CRL-MWCNTs/GA/SAF (~65.1 nm) (Figure 4d). Hence, the computational data illustrated that visualization of wet latent fingerprints was possible using the safranin-tinted CRL nanoconjugates reagent, largely due to the presence of 1 and 2 hydrogen bonds that formed between CRL-MWCNTs and GA as well as between the GA and SAF, respectively.

### 2.5. Variations in the Physico-Chemical Parameters of the Natural Outdoor Pond Water

**Figure 6:**
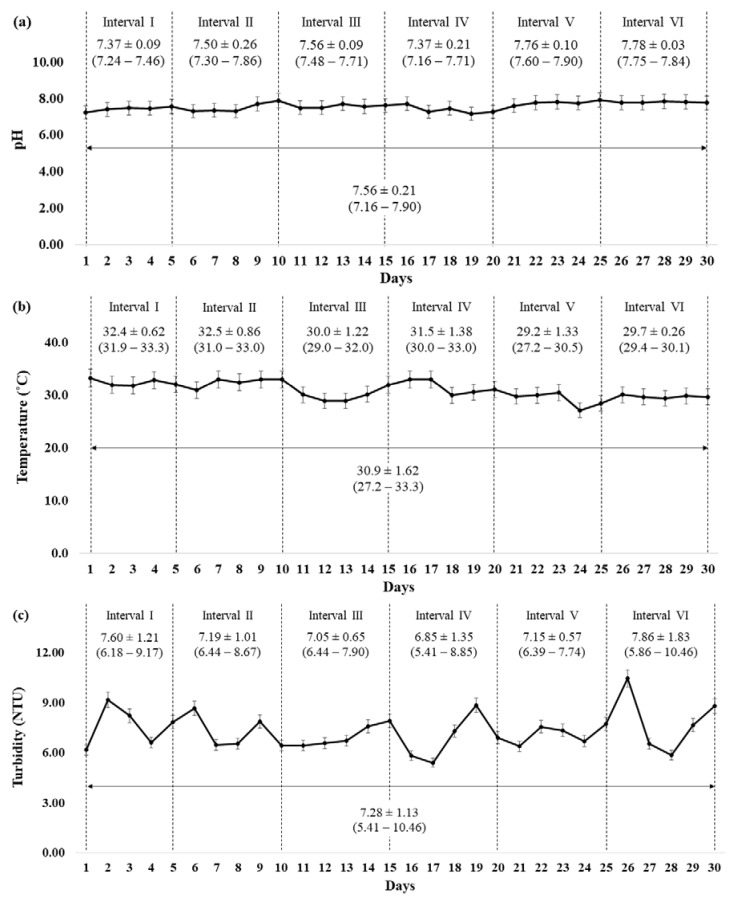

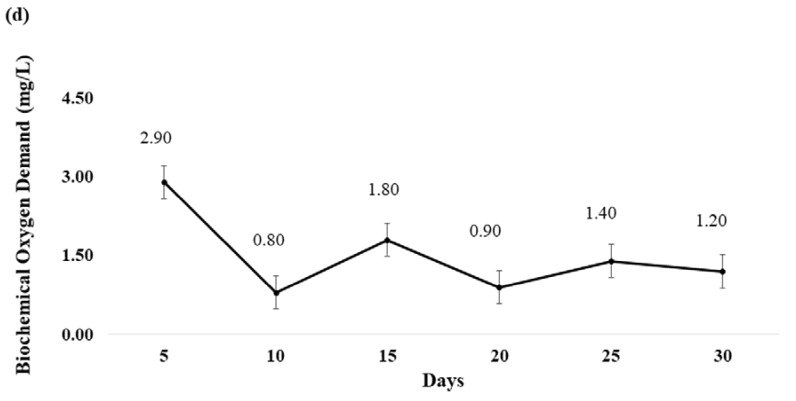
(**a**) The overall pH of the natural outdoor pond water during the 30 days of immersion period. The data are presented as mean ± standard deviation while data in the parentheses () indicate the range values; (**b**) The overall maximum daily ambient temperature of the natural outdoor pond water during the 30 days of immersion period. The data are presented as mean ± standard deviation while data in the parentheses () indicate the range values; (**c**) The overall turbidity of the natural outdoor pond water during the 30 days of immersion period. The data are presented as mean ± standard deviation while data in the parentheses () indicate the range values; (**d**) The overall biochemical oxygen demand of the natural outdoor pond water during the 30 days of immersion period. The data are presented as single values obtained during every 5-day interval of analysis.

Variations observed in the physico-chemical parameters (pH, temperature, turbidity, and biochemical oxygen demand (BOD)) of the natural outdoor pond water quality may be due to external factors such as rain, dissipation of minerals from soil, as well as human activities [46]. These variations may influence the degradation of latent fingerprints on objects immersed in water, causing difficulty in their visualization for forensic identification. In consideration of such influences, the four important physico-chemical parameters, i.e., pH, temperature, turbidity, and BOD were recorded in the present study, and the data are presented in Figure 6a–d, respectively. 

Observations revealed that the overall pH of the natural outdoor pond water during the 30 days of immersion period ranged 7.16–7.90 (mean: 7.56 ± 0.21) (Figure 6a). This signified that relatively small variations in the pH with the water remained generally neutral. Although the study found that the means of maximum daily ambient temperature (Figure 6b) during the first four immersion periods (Day 5, 10, 15, and 20) ranged 30.0–32.5 °C, lower means were recorded both during Day 21 to 25 (29.2 °C) and Day 26 to 30 (29.7 °C). The recorded mean of the maximum daily ambient temperature during the 30 days of immersion period was 30.9 ± 1.62 °C (range of means: 29.2–32.5 °C) (Figure 6b). The value is in concurrence with the typical Malaysian ambient temperature reported by the Malaysian Meteorological Department [47]. In addition, a considerably higher turbidity in the natural outdoor pond water was recorded during Day 26 to Day 30 of immersion (mean: 7.86 ± 1.83; range: 5.86–10.46 NTU) than that observed for the previous five immersion periods (Figure 6c). Such a condition may have been caused by the mechanical effect of ‘prolonged unidirectional winds’ observed during that period (Day 26 to 30). This may have increased the wave energy of the water leading to an increase in turbidity [48]. Additionally, the recorded BOD values fluctuated considerably (range 0.80–2.90 mg/L) over the 30 days of the immersion period, with the highest values recorded during the first five days (Figure 6d). As the water of the natural outdoor pond was vulnerable to variations in the microbial community [46] and organic matter from the environment [46,49], considerable fluctuations in the BOD values were expected. In short, despite the greater fluctuations in the BOD values, the fingerprints on knives immersed in the natural outdoor pond water were considered exposed to relatively small variations in pH, temperature, and turbidity.

## 3. Materials and Methods

### 3.1. Experimental Design

This research utilized 12 acetone-cleaned stainless steel (Giacomo: BL C118-7 3″) paring knives (metallic silver stainless steel throughout) as the study materials. The groomed full fingerprints (i.e., right thumbprints) were deposited over six different immersion periods (i.e., 5, 10, 15, 20, 25, and 30 days). The donor was instructed to touch its forehead and nose areas to mimic the natural action of touching such areas. Notably, the surface of the stainless steel knife used in the investigation was not uniform throughout its length and, accordingly, did not permit the use of split mark analysis. Typically, split-mark studies are used for “difficult to bisect substrates such as glass, ceramics and laminates”, in which two pieces of substrate are placed “side-by-side” to obtain groomed split fingerprints [50], Although the use of three to five different donors was suggested for a phase 1 project (proof-of-concept investigation of novel fingerprint detection methods), the guidelines provided by the International Fingerprint Research Group (IFRG) ‘are not meant to be prescriptive’ [50]. The study specifically used groomed fingerprints from a female donor because human females typically secrete fewer sebaceous constituents than males [4]. In this regard, when the safranin-tinted CRL nanoconjugates reagent reacted with the lipid constituents of fingerprints left behind by the female donor, the study expected that better visualization would be obtained for fingerprints from a male donor. Moreover, because the study was a starting point to evaluate the effectiveness of safranin-tinted CRL nanoconjugates reagent to visualize wet latent fingerprints, three separate groomed full fingerprints from a female donor were sufficient for each of the experiments. All three groomed full fingerprints were placed on the handle of each knife.

Two knives were used for each immersion period; one was used for the visualization of latent fingerprints using the safranin-tinted CRL nanoconjugates reagent, while the other used dark SPR (Sirchie, Youngsville, NC, USA). All the knives which contained fingerprints were immediately immersed for specific immersion periods in a natural outdoor pond located within Universiti Teknologi Malaysia (UTM), Johor Bahru campus (1°33′51.6″ N, 103°39′16.7″ E). To minimize interruption of the fingerprints by crawling fauna (such as aquatic snails) at the bed of the pond while allowing for the mechanical effect of the pond water, each knife was immersed in an upright position and attached to a floating Styrofoam block. Using a raffia string, two knives were tied separately on the left and right edges of a rectangular Styrofoam block (20 cm × 5 cm × 3 cm) that was secured by another raffia string to a wooden stick at the bank of the pond. The knives were then immersed at a similar depth (about 30 cm) in the water, approximately 1 m away from the edge of the natural outdoor pond. 

Because the biotic (BOD) and abiotic (pH, temperature, and turbidity) factors of the natural outdoor pond may potentially influence the quality of the visualized fingerprints, the parameters were also recorded daily around noon. BOD was analyzed at every five-day interval in the laboratory, following the method prescribed by Boyd [46]. Turbidity of the water was recorded every day in situ using a handheld pH meter (Eutech pH 5+, Thermo Fischer Scientific, Vernon Hills, IL, USA) and a turbidity meter (TN100, Thermo Fischer Scientific, Göteborg, Sweden), respectively. 

Upon completion of a specific immersion period, the two groups of knives bearing latent fingerprints were immediately subjected to either safranin-tinted CRL nanoconjugates reagent or SPR; both were observed under direct white light. The quality of the visualized fingerprints using both methods was assessed using the m-CAST grading scheme [23]. To evaluate the identifiability of the fingerprints for forensic practical casework, the visualized fingerprints were submitted to the Central Criminal Registry for Malaysia and Singapore located at the RMP Headquarters in Bukit Aman for comparison. FESEM and computational chemistry technique were used in the characterization of the visualized fingerprints using the safranin-tinted CRL nanoconjugates reagent. The collected data were used to propose the possible chemical interactions that enabled visualization of the latent fingerprints.

### 3.2. Preparation of the CRL-Multiwalled Carbon Nanotubes Solution

Purification, acid functionalization of MWCNTs, and immobilization of CRL onto the functionalized nanosupports was performed, following the methods prescribed by previous studies [20] with minor modifications. The commercial CRL (triacylglycerol ester hydrolysis EC 3.1.1.3) (type VII with measured activity of 700 U·mg^−1^), raw MWCNTs, and SAF were purchased from Sigma Aldrich (Tokyo, Japan), whereas acetone, HCl (37%), HNO_3_ (65%), H_2_SO_4_ (95–97%), phosphate buffer (pH 7), and GA (25%) were procured from QRëC (Rawang, Selangor, Malaysia). Ultra-pure water was obtained from Milli-Q^®^ Direct 8 water purification system (Merck Millipore, Darmstadt, Germany) and used without purification.

#### 3.2.1. Purification and Acid-Functionalization of MWCNTs

The raw MWCNTs (1 g) were refluxed in a round-bottom flask (100 mL) containing 4 M HCl (60 mL) with continuous stirring at 100 °C for 24 h. Once the mixture was cooled to room temperature, ultra-pure water was used repeatedly to wash all the residual acids in the purified-MWCNTs (P-MWCNTs) via centrifuge at 6000 rpm (3461× *g*) for 5 min until it reached neutrality. Upon decanting the supernatant, the P-MWCNTs were dried in a drying oven at 60 °C overnight.

The P-MWCNTs were acid-functionalized at 100 °C (with stirring) in a mixture of 6 M HNO_3_ and H_2_SO_4_ (1:3) for 6 h. It was then left to cool overnight at room temperature. After decanting the supernatant, ultra-pure water was used repeatedly to wash all the residual acids in F-MWCNTs via centrifuge at 6000 rpm (3461× *g*) for 5 min until it reached neutrality. After removing the supernatant, the F-MWCNTs were then dried in a drying oven at 80 °C overnight.

#### 3.2.2. Immobilization of CRL onto the F-MWCNTs

The commercial lipase from *C. rugosa* (75 mg) was partly purified using phosphate buffer (pH 7, 25 mL) by continuous stirring for 30 min. Upon centrifugation (6000 rpm (3461× *g*) 15 min, 4 °C), the supernatant was used for immobilization with the F-MWCNTs (50 mg). The mixture was incubated at 20 °C for 3 h, with constant magnetic stirring at 150 rpm. The unbound lipase was washed off with phosphate buffer three times. The produced CRL-MWCNTs solution were stored at 4 °C until further use.

### 3.3. Application of the Safranin-Tinted CRL Nanoconjugates Reagent for Visualizing Wet Latent Fingerprints

Upon rinsing with ultra-pure water, the CRL-MWCNTs solution (3 mg/mL) was sprayed onto the latent fingerprints, incubated for 3 min, and rinsed gently to remove the excess of CRL-MWCNTs solution. Subsequently, 5 mL of GA in 10 mL of phosphate buffer (pH 7) solution was sprayed onto the CRL-MWCNTs-treated fingerprints, followed by incubation for another 3 min, with the excess GA solution removed gently by rinsing. Upon spraying with SAF solution (50 mg/mL), the fingerprints were further incubated (3 min), followed by a gentle rinsing to remove any excess SAF solution. All the incubation steps were performed at room temperature (about 24 °C), and ultra-pure water was used during all the rinsing steps. Separate conventional pump spray bottles were used for spraying the different solutions, i.e., CRL-MWCNTs, GA, and SAF. Inclusive of spraying, rinsing, and incubating, the overall duration required for applying the prepared safranin-tinted CRL nanoconjugates reagent for visualizing wet latent fingerprints on each knife was about 10 min.

### 3.4. Assessment of Fingerprint Quality

Using a digital camera (D60, Nikon, Tokyo, Japan), the visualized fingerprints were recorded prior to tape-lifting them using a fingerprint lifting tape (SPEX Forensics, Edison, NJ, USA). The lifted fingerprints were then compared with the m-CAST grading scheme suggested by previous researchers [23]. Following the use of safranin-tinted CRL nanoconjugates reagent, the quality of the lifted fingerprints was then compared with that of SPR.

### 3.5. Characterization of the Visualized Fingerprints Using the Safranin-Tinted Nanoconjugates Reagent

Using a FESEM (SU8020, Hitachi, Tarrytown, NY, USA) operating at an accelerating voltage of 5 kV and an electric current of 10 µA, the morphology of a fingerprint, which had been placed on a glass cover slip and visualized using the safranin-tinted CRL nanoconjugates reagent, was analyzed. The sample was mounted on double-sided carbon tape on the FESEM stab, prior to sputter-coating it with a thin film of platinum.

### 3.6. Computational Chemistry for Identifying the Chemical Interactions

Calculations of the molecules were performed using the density functional theory by Gaussian09 software (G09 Academic Site License for Binary Code and TCP Linda 8 Academic Site License for x86_64-based/Linux), purchased by UTM. Geometry optimizations were undertaken using B3LYP/6-31G basis set without any symmetry constraints and simplification effects. Possible basis set superposition error was also considered. The method was used to describe the hydrogen bonding interactions, offering good performance for treating hydrogen bonds. The information obtained through this computational approach was used to provide understanding about the intermolecular structures and energies of the molecules by modelling prediction.

### 3.7. Statistical Analysis

IBM SPSS version 20.0 software was used to compare the quality of visualized fingerprints using the safranin-tinted CRL nanoconjugates reagent at specific periods of immersion with those of the prevailingly used SPR. The normality of data was tested using Kolmogorov–Smirnov and Shapiro–Wilk tests. The non-parametric Mann–Whitney *U* test was used to compare the differences in medians between the safranin-tinted CRL nanoconjugates reagent and SPR-visualized fingerprints. A level of significance of 0.05 was used to determine the significant differences between the two groups.

## 4. Conclusions

In conclusion, the safranin-tinted CRL nanoconjugates reagent produced good quality and contrast of visualized fingerprints that were comparable with SPR, even at an immersion period of 15 days. Despite a slight reduction in quality (m-CAST grade: 4) during the final three immersion periods, the fingerprints visualized using the safranin-tinted CRL nanoconjugates reagent remained forensically identifiable (m-CAST grade: 3). As a relatively safer and biodegradable method than that of the prevailing method of SPR, this safranin-tinted CRL nanoconjugates reagent is a promising, greener alternative to enhance latent fingerprints on immersed non-porous objects. Descriptions about the chemical interactions provided via the computational chemistry approach may serve as a turning point for further improvements in reagent efficiency. Although the IFRG has suggested the use of at least three different donors for a phase 1 (proof-of-concept) study, the suggestion was not prescriptive [48]. In this context, groomed fingerprints from a single female donor were used as a starting point of the investigation. The study successfully showed that this newly developed safranin-tinted CRL nanoconjugates reagent could visualize the latent fingerprints with comparable quality to that of SPR. Therefore, further studies which utilize a larger number of natural fingerprints may better elucidate the potential of the developed biotechnologically based reagent for crime scene investigations. In addition, the total duration required for visualizing latent fingerprints using this safranin-tinted CRL nanoconjugates reagent was about 10 mins, upon completion of its preparation. In consideration of this potential, further studies are required to minimize the duration for preparing and applying this reagent for visualizing latent fingerprints. Optimization of this newly developed safranin-tinted CRL nanoconjugates reagent using RSM may also be useful to enhance its capability to visualize latent fingerprints immersed in various aquatic environments.

## 5. Patent

This work has been filed for patent in Malaysia under invention disclosure number of IP/PT/2017/0025.

## Figures and Tables

**Table 1:** The quality of visualized fingerprints on stainless steel knives immersed in a natural outdoor pond over specific immersion periods using the modified-Centre for Applied Science and Technology grading scheme.

Visualization Methods	Immersion Periods (Days)						
	0	5	10	15	20	25	30
safranin-tinted CRL nanoconjugates reagent	4	4	4	4	3 *	3	3
Small particle reagent	4	4	4	4	4	3	3

Mann-Whitney *U* test was used for comparing the differences in medians between the safranin-tinted CRL nanoconjugates reagent and Small Particle Reagent groups. For each group and immersion period, three replicates of fingerprints from a single donor were used and the data are presented as median. The symbol (*) indicates the significant difference in the quality of fingerprints visualized using the safranin-tinted CRL nanoconjugates reagent with that of SPR (*p* < 0.05).

## Abbreviations

SPR	Small particle reagent
CRL	*Candida rugosa* lipase
MWCNTs	Multi-walled carbon nanotubes
GA	Glutaraldehyde
SAF	Safranin T dye
m-CAST	Modified-Centre for Applied Science and Technology
BOD	Biochemical oxygen demand
RMP	Royal Malaysia Police
RSM	Response surface methodology
FESEM	Field emission scanning electron microscopy
F-MWCNTs	Functionalized-multi-walled carbon nanotubes
UTM	Universiti Teknologi Malaysia
IFRG	International Fingerprint Research Group
